# Leniency and halo effects in marking undergraduate short research projects

**DOI:** 10.1186/1472-6920-4-28

**Published:** 2004-11-29

**Authors:** Brian H McKinstry, Helen S Cameron, Robert A Elton, Simon C Riley

**Affiliations:** 1 Community Health Sciences, University of Edinburgh, Edinburgh, Scotland, UK; 2 Medical Teaching Organisation, College of Medicine and Veterinary Medicine, University of Edinburgh Scotland, UK; 3 Obstetrics and Gynaecology Section, Centre for Reproductive Biology, University of Edinburgh, Scotland, UK

## Abstract

**Background:**

Supervisors are often involved in the assessment of projects they have supervised themselves. Previous research suggests that detailed marking sheets may alleviate leniency and halo effects. We set out to determine if, despite using such a marking schedule, leniency and halo effects were evident in the supervisors' marking of undergraduate short research projects (special study modules (SSM)).

**Methods:**

Review of grades awarded by supervisors, second markers and control markers to the written reports of 4^th ^year medical students who had participated in an SSM during two full academic years (n = 399). Paired t-tests were used to compare mean marks, Pearson correlation to look at agreement between marks and multiple linear regression to test the prediction of one mark from several others adjusted for one another.

**Results:**

There was a highly significant difference of approximately half a grade between supervisors and second markers with supervisors marking higher. (t = 3.12, p < 0.01, difference in grade score = 0.42, 95% CI for mean difference 0.18–0.80). There was a high correlation between the two marks awarded for performance of the project and the written report by the supervisor (r = 0.75), but a low-modest correlation between supervisor and second marker (r = 0.28). Linear regression analysis of the influence of the supervisors' mark for performance on their mark for the report gave a non-significant result. This suggests a leniency effect but no halo effect.

**Conclusions:**

This study shows that with the use of structured marking sheet for assessment of undergraduate medical students, supervisors marks are not associated with a halo effect, but leniency does occur. As supervisor assessment is becoming more common in both under graduate and postgraduate teaching new ways to improve objectivity in marking and to address the leniency of supervisors should be sought.

## Background

There is compelling evidence from the literature that supervisors may be unreliable when asked to assess the performance of their own students. Effects such as the so-called 'halo' effect [1] in which a good or bad performance in one area affects the assessor's judgement in other areas and 'leniency'[2] where assessors are reluctant for a variety of reasons including fear of impairing the student-teacher relationship, fear of a negative emotional reaction from the student, or of poor reflection on the teacher's own expertise may come into play when assessing students' work. Increasingly however, particularly in medical education, teachers and supervisors are being asked to assess their own students. We describe a study to investigate to what extent effects such as halo and leniency were operating in supervisor marked Special Study Modules (SSMs) in the Edinburgh University undergraduate course.

SSMs were introduced into the fourth year of the 5-year undergraduate medical curriculum in 1995. This was in response to the recommendations from the General Medical Council's document Tomorrow's Doctors [3]. Edinburgh SSMs aim to develop students' skills in self-directed and enquiry-led learning, team working and writing a short thesis or report (of about 3000 words). The development also gives students an opportunity to choose an area of study and to pursue it in depth. Students spend 8 weeks on individual projects under the supervision of a member of the University of Edinburgh academic staff working on a wide range of projects in virtually every specialty including clinical audit, laboratory-based research and clinical projects, with over 300 supervisors involved.

**Figure 1:**
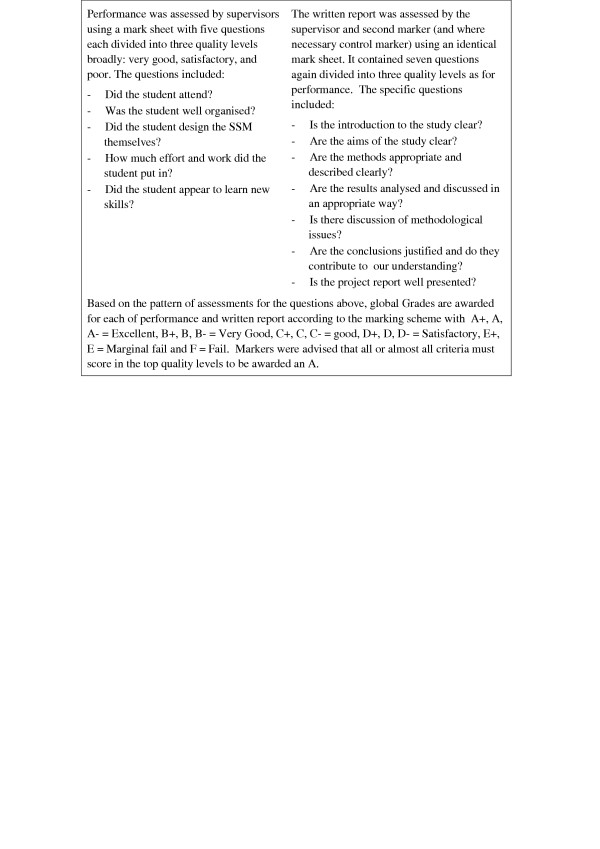
Marking scheme

For assessment an identical structured form was used by all assessors. Supervisors were asked to assess students on both their performance during the 8-week SSM and on their written report. Each component was awarded a separate grade by the supervisor and a combined grade for both was calculated by taking the mean grade. This mean grade contributed 50% to the final SSM mark. A second marker, usually another SSM supervisor working in a related area of research, with no prior knowledge of the student or the project would also assess the written report and this mark contributed 50% of the final mark. It was intended that this would permit the supervisors to be able to compare their own students' projects with others and would ensure greater consistency in the marking. Where there was a discrepancy of more than one full alphabetic grade category (e.g. A and C) between the supervisor and the second marker or where a fail grade was awarded, the report was assessed without prior knowledge of previous marks by at least one other experienced member of the Board of Examiners (control marker). The mark schemes for these assessments are described in Figure 1. Other than the guidance described there is no formal training of assessors.

On reviewing the marks we noticed that there appeared to be a high correlation between the supervisor's marks for any one student's performance during the attachment and marks for their written report but a low correlation between the supervisor's and second marker's marks for the student's written report. This observation led us to investigate the hypothesis that the supervisors' knowledge of the students influenced their mark for the written report.

## Methods

We reviewed the grades of all the students from two full academic years (n = 399) who had participated in an SSM between 1999–2001 to answer the following questions: What is the correlation between the supervisor's marks for performance and report, and if this is high is there a causal relationship? Is there a real difference in the marks awarded for the report between the supervisor and the second marker, and if so what is the cause of the difference? In cases of discrepant marks where the reports were further marked by control markers; what is the correlation between the control markers with the supervisors' and second markers?

The grades awarded for Performance and Reports were translated to a numerical scale thus: A+ = 1, A = 2, A- = 3, B+ = 4, through to E = 14. No grades below E (Marginal Fail) were awarded.

We used paired t-tests to compare mean marks, Pearson correlation for looking at agreement between markers, and multiple linear regression to test the prediction of one mark from several others adjusted for one another.

## Results

Table 1 shows the mean and standard deviation expressed in a numerical scale of grades given by supervisors, second markers, and control markers.

**Table 1:** Mean and standard deviation of grades expressed on a numerical scale (grade score) awarded by the supervisor for performance and for the written report, and by the second marker and control markers for the written report (A+ = 1, A = 2 etc.; the lower the grade score the higher the mark)

Marker	Component marked	N	Grade score	Standard deviation
Supervisor	Performance	383	4.12	2.60
	Written report	383	4.64	2.50
	**Combined mark**	389	4.45	2.48
Second marker	Written report	373	5.16	2.40
Mean of Control markers	Written report	98	5.86	**2.21**
	**Final mark**	399	5.18	2.06

Using paired t-tests to compare mean marks for the written report between supervisors and second markers revealed a highly significant difference (t = 3.12, p < 0.01), with the supervisor scoring higher than the second marker (difference in grade score = 0.42, 95% confidence interval for mean difference 0.18 – 0.80). Correlation between the two marks was modest, r = 0.28. Control markers tended to mark the lower scoring students. While there was a numerical difference (lower) between control marks for the written report and the supervisor this failed to reach significance (t = 1.81, p = 0.07). Despite there being no significant difference between control markers and second markers, correlation was low (r = 0.11).

There was considerably higher correlation between the two marks awarded by each supervisor i.e. for the students' performance and written report r = 0.75 but again there was a highly significant difference in the mean marks t = 5.69, P < 0.001 (difference in grade score = 0.52; 95% confidence interval for mean difference 0.34 – 0.69)

Analysis of the influence of the supervisor's mark for performance on his/her mark for the report was done by linear regression. This gave a non-significant result for the performance mark adjusted for the written mark. Table 2 summarises these comparisons.

**Table 2:** Summary of statistical analysis of data

	Supervisor Written Report	Control Marker Written Report
Supervisor Performance	t = 5.69, p < 0.001. Performance scoring higher than report (difference in grade score = 0.52) Highly significant difference. r = 0.75 Linear regression – non-significant result	t = 3.07, p = 0.003. Significant difference

Second Marker Written Report	t = 3.12, p < 0.01 Highly significant difference. Supervisor scoring higher than second marker (difference in grade score = 0.42, 95% confidence interval for mean difference 0.18 – 0.80). r = 0.28	t = 0.68 No significant difference. r = 0.11

Control Marker Written Report	t = 1.81, p = 0.07 No significant difference.	

## Discussion

Analysis of the grades awarded demonstrated that there is a significant difference in the mean marks awarded by the supervisors and second markers, with the supervisors marking nearly half a grade higher than the second markers. The correlation was also modest between these markers' assessments of the reports suggesting that the two groups of markers were not using the same criteria to reach their decision, despite being provided with descriptors and a mark scheme. It is important to note that most supervisors were also second markers. At the same time they were assessing their own students' project, and so had a direct and simultaneous comparison. Therefore, the same individual appeared to use different criteria depending on whether they marked their supervised student's report or others. The lack of significant difference between the mean marks awarded by the second marker and the control marker suggests that they were awarding the same range of grades overall but the modest correlations indicate that in the case of individual students there was again significant inter-marker variability. Control markers, unlike supervisors and second markers (who may only supervise one project a year) have experience of reviewing large numbers of SSM reports. There was also a significant difference in the mean marks awarded by supervisors for performance and for written reports but in this analysis there was a much higher correlation between the marks. However, further analysis of this finding by linear regression failed to demonstrate an undue influence of the performance mark on that of the report.

Although we have been unable to provide evidence that the supervisor's mark for performance has an undue influence on the mark for the written report (halo effect), we have demonstrated that the supervisors mark significantly higher than second markers, suggesting a leniency effect. This indicates that the supervisor's mark is influenced by having known and worked with the student. Such effects have been demonstrated before in many forms of education [4-8]. Some of the factors contributing to this may include insight and therefore sympathy for the student's difficulties in performing the project; inability to be objective when the student has become part of the work team; unwillingness of the supervisor to acknowledge that a piece of work emanating from his team is poor quality, or lacking the confidence or courage to feed back personally a bad assessment to the student. These factors need further exploration.

Increasingly in medical education supervisors are expected to summatively assess their students [9,10]. Assessors are unlikely to be affected equally by leniency and halo effects and this will advantage some and disadvantage others among their students. These effects are likely to be strongest on supervisors who, like some of those in our study, are assessing a relatively small number of students and are inexperienced in assessment [6]. If we are to continue to use supervisor-based assessments we must find ways to combat these effects. Other authors' suggestions for improving objectiveness and partially overcoming halo and leniency effects include detailed marking sheets [6,11], training for assessors in providing feedback of assessments [5], and also providing feedback on assessors' marking performance [6].

We are aware that the marking scheme in Figure 1, while structured, still permitted a fair degree of interpretation by examiners. Since carrying out this project we have introduced more detailed marking schemes with specific questions and detailed descriptors for each level of achievement for assessing the students' performance and report. This now includes an assessment of how the student overcame any problems which arose and how this may have affected the outcome of the project. We have also provided more detailed guidance to markers. We intend to review the inter-marker variability in light of the increased guidance given to markers.

These findings raise the ethical question as to whether or not we should continue to utilise supervisors in this assessment process. We are planning to continue to use supervisors as markers because of the expertise they bring to the specific field of study and their realistic expectation of the difficulties encountered by the student during the course of the project. Also the supervisor is sometimes the only person capable of marking the student's performance, which we consider a very valuable assessment of the students personal and professional abilities. We do realise that this is a difficult responsibility for supervisors. Better staff development of supervisors as markers and a more detailed marking schedule may help ensure appropriate marks for performance. Furthermore, we will also consider introducing 360 degree assessment to include all members of staff who have interacted with the student, particularly to improve formative feedback to students.

## Conclusions

In this paper we have demonstrated the problem of inter-marker variability between the supervisor of undergraduate projects and the second marker even when using a mark scheme. This emphasises the difficulty in creating mark schemes and providing adequate staff training which ensures that markers apply the criteria in the same way in very varied reports. On average, supervisors awarded higher marks for their students' reports than the second markers but the influence of the performance mark on this was not significant. We would suggest that this difference is due to leniency in the supervisor resulting from the student being part of the supervisor's team, but these influences need further exploration.

## Competing interests

SR, HC and BMcK are all involved in undergraduate teaching at the University of Edinburgh

## Authors' contributions

BMcK, HC and SR contributed equally to the design of the research and the writing of the project. RE analysed the data.

## Pre-publication history

The pre-publication history for this paper can be accessed here:


